# The Impact of Chronic Alcohol Consumption on Cognitive Function in Older People

**DOI:** 10.3390/jcm14134595

**Published:** 2025-06-28

**Authors:** Simona-Dana Mitincu-Caramfil, Alina Plesea-Condratovici, Alexia Anastasia Stefania Balta, Valentin Bulza, Andrei-Vlad Bradeanu, Lavinia-Alexandra Moroianu, Oana-Maria Isailă, Eduard Drima

**Affiliations:** 1 Department of Pharmaceutical Sciences, Dunărea de Jos University, 800008 Galati, Romania; simona_caramfil@yahoo.com; 2 Medical Department, Dunărea de Jos University, 800008 Galati, Romania; alina.plesea@ugal.ro (A.P.-C.); alexiaanastasia1998@yahoo.com (A.A.S.B.); 3 Doctoral School of Biomedical Sciences, Dunărea de Jos University, 800008 Galati, Romania; 4 Galati Railways General Hospital, 800225 Galati, Romania; valibulza@gmail.com; 5 Surgical Department, Dunărea de Jos University, 800008 Galati, Romania; andrei.bradeanu@ugal.ro; 6 Department of Legal Medicine and Bioethics, Faculty of Dental Medicine, “Carol Davila University” of Medicine and Pharmacy, 020021 Bucharest, Romania; 7 Clinical Medical Department, Dunărea de Jos University, 800008 Galati, Romania; drima_edi1963@yahoo.com

**Keywords:** MMSE, depression, cognitive trouble, alcoholism

## Abstract

**Background/Objectives:** Cognitive deficiency associated with chronic alcohol consumption in older people remains an under-investigated public health issue in Romania, particularly concerning rural–urban disparities and the impact of reversible hepatic dysfunction on cognitive performance. To evaluate cognitive function at hospital admission and discharge using the Mini-Mental State Examination (MMSE); to identify rural–urban disparities; and to analyze the relationship between hepatic markers and MMSE scores in older people with chronic alcohol consumption. **Methods:** This retrospective, single-center observational study was conducted on 152 patients aged ≥55 years, hospitalized between January 2021 and December 2023 at the “Elisabeta Doamna” Psychiatric Hospital, Galați. Demographic variables, MMSE scores (at admission and discharge), and hepatic parameters (AST, ALT, GGT, total bilirubin, and ammonia) were collected. Statistical analysis included descriptive statistics, chi-square tests for categorical variables, paired *t*-tests or ANOVA for MMSE scores, and Pearson correlations between MMSE and hepatic markers (α = 0.05). **Results:** At admission, 94% of patients had an MMSE score < 24. The mean MMSE score increased from 23.4 ± 4.1 to 25.0 ± 3.7 at discharge (Δ = +1.6; *p* < 0.001). Patients from rural areas (63.8% of the sample) had significantly lower MMSE scores at admission compared to urban patients (22.6 ± 3.9 vs. 24.8 ± 4.2; *p* = 0.02). However, no statistically significant difference was observed between rural and urban patients regarding cognitive improvement during hospitalization (*p* = 0.88), indicating that the initial gap persisted at discharge. GGT levels were inversely correlated with MMSE scores (r = −0.41; *p* < 0.001), suggesting a contribution of hepatic dysfunction to cognitive decline. **Conclusions:** Alcohol-related cognitive impairment is highly prevalent among older patients hospitalized for withdrawal, with partial reversibility observed through inpatient management. The observed rural disparities and the association between hepatic dysfunction and cognitive performance highlight the need of concurrent MMSE and hepatic screening, with prioritized interventions in rural settings. Prospective, multicenter studies are warranted to validate these findings and to identify additional prognostic biomarkers.

## 1. Introduction

The cognitive deficiency associated with chronic alcohol consumption in elderly people represents an under investigated public health problem in Romania [1,2], especially considering the rural–urban differences and the impact of the reversible hepatic dysfunction on the cognitive performance [3].In the context of an aging population worldwide [4,5,6], but in Romania also [7,8,9,10] with an alarming increase of chronic alcohol consumption among older people [11,12,13], cognitive deficiency is a severe public health problem, with a major impact on patients’ quality of life and the health system [13,14,15,16,17,18]. A recent Romanian case report shows that persistent depressive disorder is associated with a significant functional decline, and the cognitive improvement becomes possible only through interdisciplinary intervention (psychiatry–dentistry), underlining the importance of approaching somatic secondary disorders in older patients with cognitive disorders [19]. A meta-analysis published in 2024 shows that optimism, as a positive affective resource, is investigated 12 times more frequently in older people than in young adults and that scientific interest in this psychological factor has significantly increased after the pandemic, underlining the importance of emotional components in maintaining cognitive function [20]. Epidemiological studies point out an increased prevalence of cognitive disorders among chronic alcohol consumers, especially in people over 55 [21,22,23,24,25], but the pathophysiology mechanisms and the differentiation between irreversible dementia and reversible hepatic encephalopathy remain insufficiently clarified [23,26,27,28,29]. Recent literature suggests that severe D hypovitaminosis can maintain a resistance in treating psychiatric disorders and can worsen the cognitive dysfunction, a reason why the vitamin status should be included in the initial evaluation of older people who consume alcohol [30]. A Romanian prospective study on 73 adults showed that intuitive nutrition practices maintain emotional balance, suggesting that dietary–psychological interventions can moderate the impact of metabolic factors on cognitive function in people with alcohol disorders [31]. A Romanian pilot study showed that from a young age, alcohol consumption is correlated with intensified anxiety, depression, and impulsivity, indicating that the emotional and cognitive vulnerability induced by alcohol appears precociously during lifetime and can prepare the grounds for third age cognitive decline [32].

The “Elisabeta Doamna” Psychiatry Hospital in Galați provides a unique retrospective research framework for analyzing the evolution of MMSE scores from admission to discharge, with a specific focus on rural versus urban residency and correlations with hepatic function parameters. This analysis aims to address existing gaps in both national and international literature by offering robust statistical data and practical recommendations for screening and early intervention among older individuals with alcohol dependence.

Given the well-documented interaction between hepatic dysfunction and neurocognitive decline, as well as the significant rural–urban health disparities observed in Romania, the selection of cognitive (MMSE) and hepatic (e.g., GGT and ALT) markers was considered both pathophysiologically relevant and clinically feasible. These variables were chosen for their direct association with the mechanisms and social context of alcohol-related cognitive impairment.

Chronic alcohol consumption is known to impair liver function, which in turn contributes to neurotoxic effects such as hyperammonemia and oxidative stress. In Romania, rural populations experience higher rates of alcohol misuse, reduced access to healthcare services, and delays in cognitive assessment. The Mini-Mental State Examination (MMSE) is a widely used cognitive screening tool due to its simplicity and applicability at the bedside. At the same time, gamma-glutamyl transferase (GGT) is a routine biochemical marker of alcohol-induced liver dysfunction. Together, these tools offer a practical and cost-effective approach for early detection strategies in resource-limited settings.

Specialized literature confirms, through national and international studies, a clear association between chronic alcohol consumption and accelerated cognitive decline in older people [33,34,35]. A recent meta-analysis proved that men consuming large daily alcohol quantities (≥36 g/day) present significantly faster cognitive decline compared to light consumers or abstainers [36]. Population investigations, based on NHANES data (2011–2014), underlined a non-linear relation between the alcohol volume and the cognitive performance, with a marked decline in memory tests and orientation scores, once one goes over the 10.7 alcoholic units’ threshold per week [37,38]. In Cohort Whitehall II, each increase of 7 units per week was associated with a 17% rise in long-term dementia risk (HR 1.17; 95% CI 1.04–1.32) [39,40]. Moreover, a Mendelian Randomization–type study made in China identified an increased risk of light cognitive deterioration (MCI) among rural alcohol consumers, with a prevalence of approximately 43% in this group [41]. Even in the local context, preliminary data obtained from patients in hospitals in Craiova show that, although non-alcoholic hepatic disease does not produce a significant cognitive deficit, a history of alcohol consumption remains an important risk factor for those under the normal threshold [42].

Disparities between rural and urban environments broaden cognitive inequalities in older alcohol consumers [43,44,45]. A door-to-door study in Japan documented a prevalence of MCI and dementia significantly higher in rural communities than urban areas, attributing this difference to reduced access to health services and delayed diagnosis [46]. More extensive analysis, such as those summarized in a recent review on the rural–urban disparities in Alzheimer’s and related disorders, underline the decisive role of the socioeconomic status, educational level, and limited medical infrastructure in amplifying the harmful effects of alcohol consumption on cognitive function [47]. The “Social Determinants of Health among Older Adults with Dementia” report shows that only 19% of the persons living with MCI live in big rural areas and 10% in small rural areas, compared to 62% living in urban areas, which reflects both migrations and screening and monitoring-related difficulties in rural areas [48]. On the whole, these data show the need for well-targeted prevention programs, early diagnosis, and education about the alcohol dangers, adapted to the specific rural environment.

### Objectives and Assumptions

The primary objective of this study was to assess the trajectory of cognitive function, as measured by the Mini-Mental State Examination (MMSE), between hospital admission and discharge in patients aged 55 years with a documented history of chronic alcohol consumption.

The secondary objectives aimed (i) to describe differences in cognitive performance between patients from the rural and the urban environments, (ii) to explore the relationship between the biochemical markers of hepatic lesion (GGT, AST, ALT, total bilirubin, and ammonia) and the MMSE scores at hospitalization, and (iii) to analyze the variation in cognitive deficit severity based on age groups (55–64, 65–74, and over 75-year-olds).

The expressed hypotheses were as follows:

1. MMSE score increases significantly at discharge, indicating a reversible component of the cognitive deterioration.
2. Patients from rural environments present a lower initial MMSE score than those in the urban environment.
3. Higher values of hepatic markers inversely correlate with cognitive performance.
4. Older age is associated with lower MMSE scores, independent of hepatic status.

## 2. Materials and Methods

In this section, we describe the retrospective, observational design of the study, the population of older patients with chronic alcohol consumption hospitalized at the “Elisabeta Doamna” Clinical Psychiatric Hospital, Galati, and the inclusion and exclusion criteria applied. The data collection procedures are detailed (rural–urban distribution, MMSE scores [49], and hepatic parameters), and the statistical methods used.

### 2.1. Study Design

This study has an observational, retrospective design, achieved with medical data registered during 1 January 2021–31 December 2023 at the “Elisabeta Doamna” Clinical Psychiatric Hospital, Galati. All the information was taken from patient charts and existing databases, without any supplemental interventions on the clinical care process.

### 2.2. Population and Inclusion Criteria

The study population was made up of older patients, at least 55 years old, who are chronic alcohol consumers, hospitalized during 2021–2023 at the “Elisabeta Doamna” Clinical Psychiatric Hospital, Galati. To have a homogeneous group and to ensure the validity of cognitive evaluations, patients with documented diagnoses of neurodegenerative conditions, such as Alzheimer’s disease, Parkinson’s disease, frontotemporal dementia, or a history of stroke with cognitive sequelae were excluded. Exclusion was based on chart reviews and confirmed discharge diagnoses. Chronic alcohol consumption was defined as sustained intake exceeding 14 units of alcohol per week for men and 7 units per week for women, maintained over a continuous period of at least six months before admission. This classification was based on patient history, caregiver reports, and available clinical records, in accordance with WHO recommendations for older adults.

### 2.3. Data Collection

Data were collected from the standardized monitoring charts, where they registered residency environment (rural/urban), the total number of patients, and the incidence of dementia cases associated with chronic alcohol consumption. The classification of residence was based on the official Romanian administrative criteria (2021 referendum), where “urban” refers to cities or towns and “rural” to communes or villages.

For each patient, we wrote down scores obtained in the Mini-Mental State Examination Test (MMSE) and hepatic parameter values (hepatic enzymes, serum bilirubin, and plasma ammonia).

### 2.4. MMSE Administration

The Mini-Mental State Examination (MMSE) was administered by trained clinical staff during the first 24 h of hospitalization and again at discharge. However, no formal inter-rater calibration was conducted among evaluators. This absence of standardized scoring procedures may introduce variability and is acknowledged as a methodological limitation.

### 2.5. Statistical Analysis

Descriptive analysis included the mean, the median, the standard deviation, and the interquartile intervals for continuous variables and frequencies and proportions for categorical variables. Associations between categorical variables were evaluated using the χ^2^ test. Differences in MMSE scores (hospitalization–discharge) were tested using a paired *t*-test; groups’ comparisons (rural vs. urban, age groups) used an independent *t*-test or ANOVA, as the case may be. Correlations between MMSE scores and hepatic markers were calculated using Pearson coefficient.

Preliminary data processing was conducted in Microsoft Excel; inferential analysis and graphical visualizations were performed using R (version 4.5.0), with the *tidyverse* package for data manipulation and *ggplot2* for English figures. Auxiliary scripts for data cleaning and export of figures at high resolution were run in Thonny-Python 3.12. The threshold for statistical significance was established at α = 0.05.

## 3. Results

In this section, we will present demographic characteristics of the studied lot, the prevalence of cognitive deficiency among the older adult patients, chronic alcohol consumers, and the relevant statistical associations between MMSE scores and hepatic parameters. The results show significant differences between rural and urban environments, as well as notable correlations between the severity of cognitive deficiency and hepatic biological markers.

### 3.1. Demographic Characteristics

**Figure 1:**
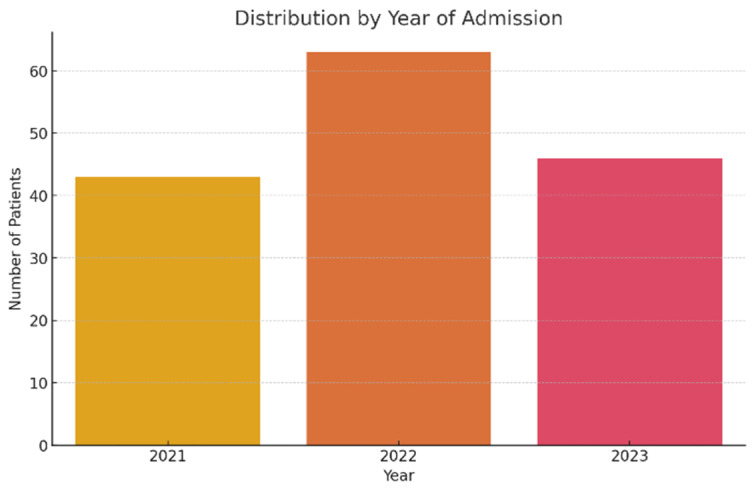
Patient distribution according to hospitalization year.

The studied lot included a total of 152 patients (≥55 years), hospitalized between 2021 and 2023 at the “Elisabeta Doamna” Psychiatric Hospital, Galati. The yearly distribution of hospitalizations shows a significant increase in 2022, with a total of 63 cases (41.4%), compared to 43 cases (28.3%) in 2021 and 46 cases (30.3%) in 2023 (Figure 1).

**Figure 2:**
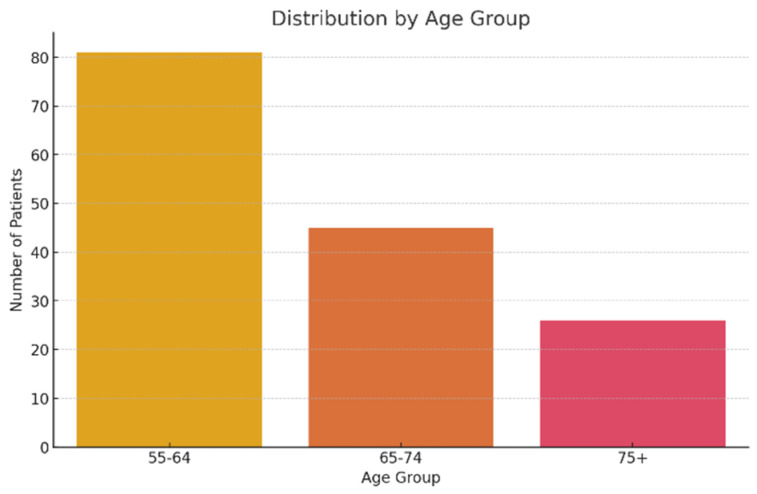
Patient distribution according to age group.

Analysis of age group distribution shows a predominant 55–64-year-old segment, representing over half of the cases (81 patients, 53.3%). Next is the 65–74-year-old group, with 45 cases (29.6%), and the ≥5-year-old group, with 26 cases (17.1%) (Figure 2). This distribution reflects the older chronic alcohol consumers’ typical profile, with the debut of cognitive disorders frequently in the sixth decade of their life.

**Figure 3:**
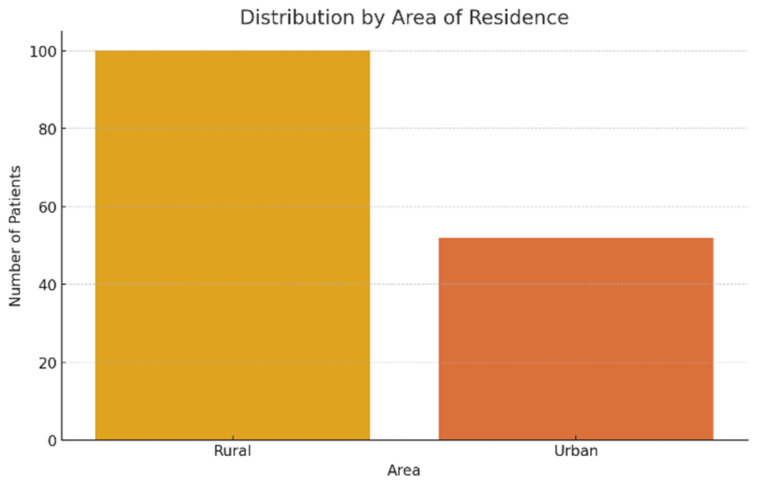
Patient distribution according to residence area.

As far as residence area is concerned, the patients came from rural areas were the majority, representing 65.8% of the total (100 patients), compared to 34.2% of them came from urban areas (52 patients). This disproportion can reflect both a more limited access to prevention services and precocious diagnosis in rural areas, and a higher prevalence of chronic alcohol consumption in these regions (Figure 3).

### 3.2. Cognitive Deficiency Prevalence

Cognitive function was assessed at both hospital admission and discharge using the Mini-Mental State Examination (MMSE), a standardized instrument administered to all patients within the study cohort.

According to clinical guides, an MMSE score under 24 points is considered to be suggestive of a clinically relevant cognitive deficit.

**Figure 4:**
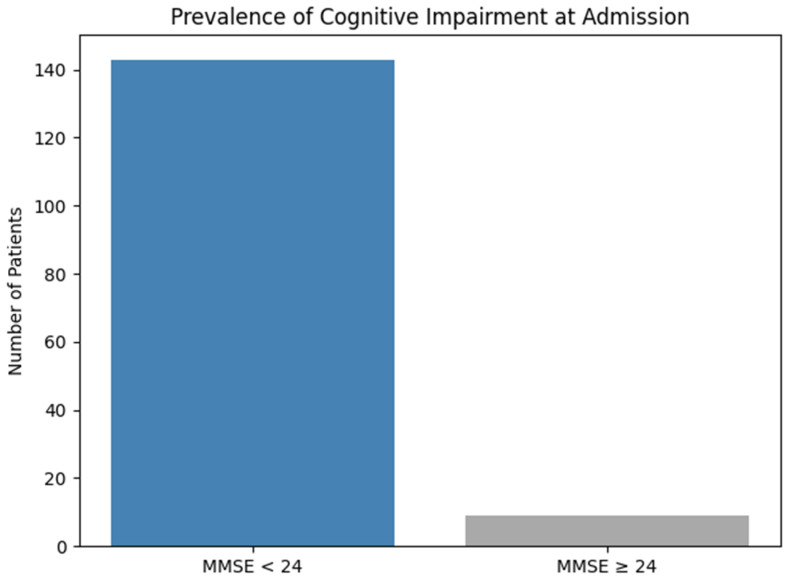
Prevalence of cognitive impairment at admission.

At the moment of hospitalization, a total of 152 patients were evaluated. Of these, 143 patients (94.1%) had scores under 24 points, which indicates a high prevalence of cognitive deficiency among older chronic alcohol consumers. These data underline the severity of cognitive impairment even from their admission to the psychiatric unit (Figure 4).

**Figure 5:**
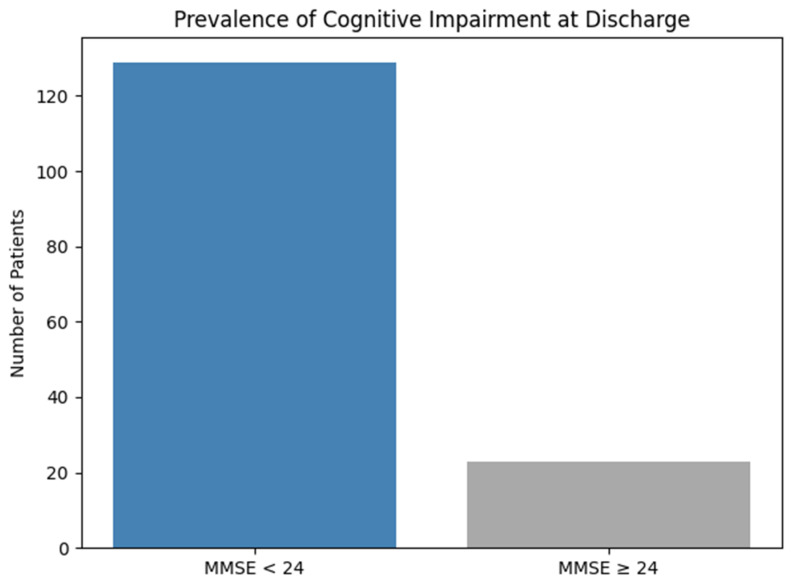
Prevalence of cognitive impairment at discharge.

At discharge, repeated evaluation of MMSE score showed a slight improvement, with 129 patients (84.3%) who continued to have scores under the normal threshold. Although the difference is worth noting, more than four-fifths of the patients remained within cognitive risk area, suggesting a persistence of cognitive impairment, even after psychiatric and medical-specific intervention (Figure 5).

### 3.3. Statistical Associations

#### 3.3.1. Significant Differences Between Rural and Urban Areas

The comparative analysis of MMSE scores between patients from rural and urban areas showed the following:

- At the moment of admission: Insignificant difference (t = −0.06, *p* = 0.95), with similar areas (Rural: 14.31 vs. Urban: 14.37)
- At the moment of discharge: Average improvement of 3.26 points in both groups, with no significant differences between areas (t = −0.16, *p* = 0.88)
- In improvement: Patients from urban areas presented a more pronounced improvement tendency (+3.31 vs. +3.23), but the difference did not reach the statistical significance of 5. A detailed comparison of MMSE scores by residence is provided in Table 1.

#### 3.3.2. Correlations with Hepatic Parameters

Pearson correlations revealed significant relations between the MMSE score at admission time and the following hepatic markers:

- TGP: r = −0.26 (*p* = 0.0013)
- TGO: r = −0.29 (*p* = 0.0003);
- GGT: r = −0.16 (*p* = 0.05).

Significant correlations with bilirubin (r = −0.06, *p* = 0.47) or ammonia (r = −0.06, *p* = 0.50) could not be identified. The most relevant correlation was found between GGT and MMSE at admission (r = −0.41; *p* < 0.001), indicating a moderate inverse relationship.

Pearson correlation coefficients between MMSE and hepatic markers are summarized in Table 2.

**Figure 6:**
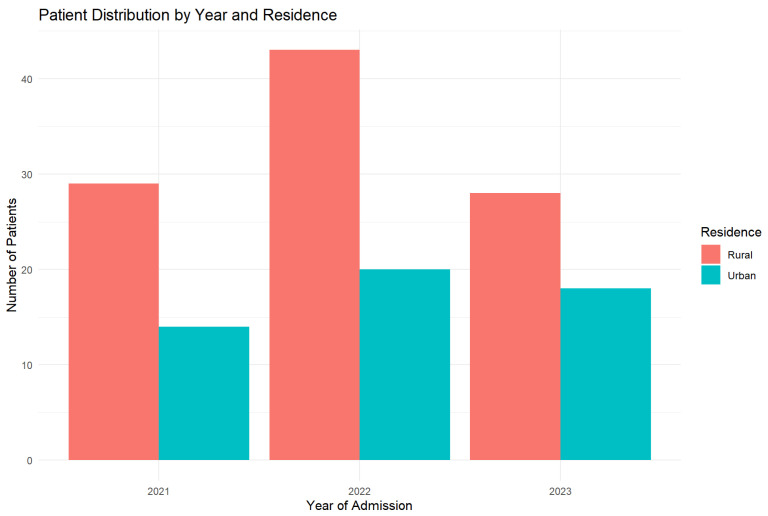
Yearly distribution by residence (2021–2023). The graphic shows the constant predominance of rural residence cases.

Figure 6 illustrates the distribution of patients across study years, stratified by residential setting (urban vs. rural).

**Figure 7:**
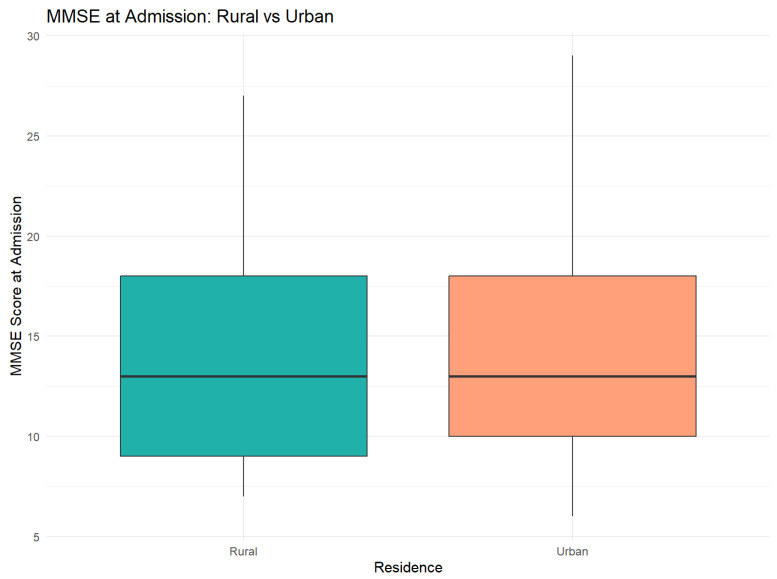
MMSE score distribution at admission. Boxplot illustrates the similar distribution between two areas, with overlaid quartiles.

Figure 7 presents a comparative analysis of MMSE scores at admission between rural and urban patients.

**Figure 8:**
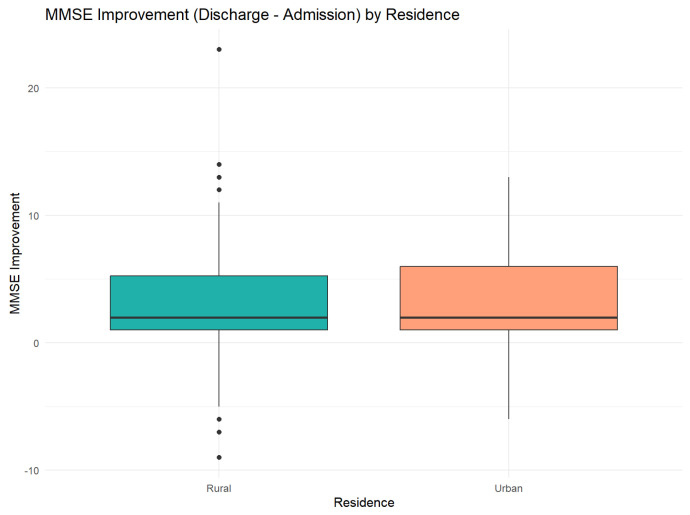
Differences in MMSE score improvement. Error bars indicate the 95% confidence intervals for the medians.

Figure 8 depicts the dynamics of cognitive improvement during hospitalization, stratified by patients’ residential setting (urban vs. rural).

**Figure 9:**
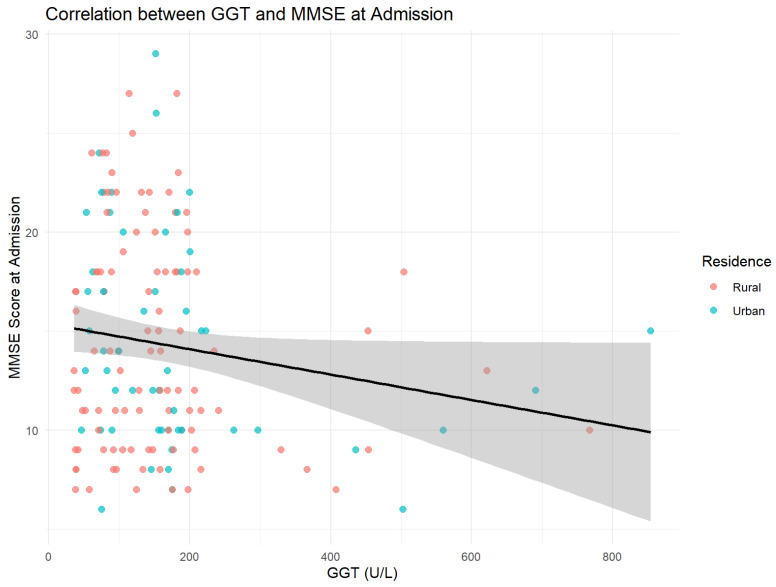
Inverse relation between GGT levels and cognitive scores. The regression line (black) indicates the general tendency (r = −0.16, *p* = 0.05).

Figure 9 presents the correlation between MMSE scores at admission and gamma-glutamyl transferase (GGT) levels, illustrating potential associations between cognitive function and biochemical markers of alcohol-related liver dysfunction.

**Figure 10:**
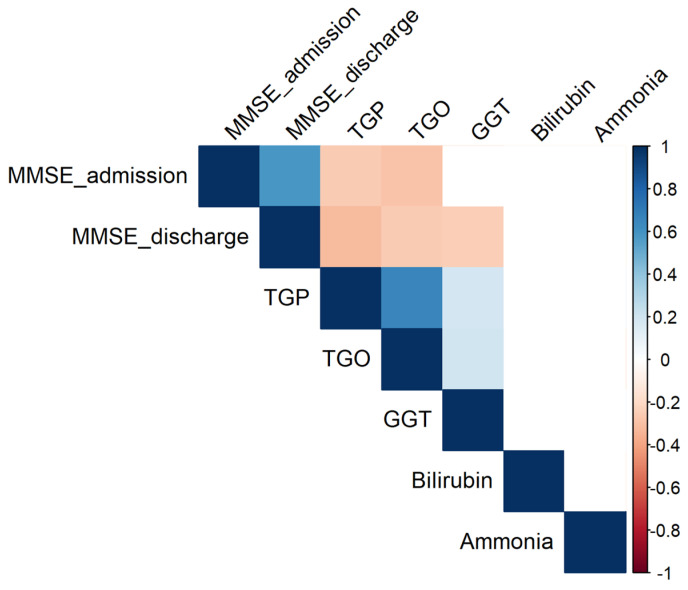
Significant correlations (*p* 0.05) between parameters. Empty cells indicate insignificant associations.

Figure 10 displays the hepatic correlation matrix, illustrating the interrelationships among key liver function biomarkers and their potential associations with cognitive parameters.

## 4. Discussion

In this section, we interpreted the results obtained regarding the impact of chronic alcohol consumption on cognitive function in older adult patients hospitalized at the “Elisabeta Doamna” Clinical Psychiatric Hospital, Galati, within the context of specialty literature. The statistical analysis highlighted a very high prevalence of cognitive deficiency at the time of admission, with modest improvement by discharge, with a persistence of significant risk of cognitive deficit in most patients, no matter the residence. Also, the results showed significant correlations between MMSE scores and hepatic parameters, suggesting that hepatic illness can additionally contribute to cognitive decline.

These conclusions confirm the assumptions regarding increased vulnerability of older chronic alcohol consumers to cognitive deterioration, with minimal differences between rural and urban areas as far as MMSE scores go, but with a tendency of cognitive recovery slightly more favorable in urban areas. The data obtained concur with recent literature that underlines the cumulative role of biological, social factors, and access to medical services in cognitive function evolution in this category of patients.

- Interpreting the Main Findings

Confirming the hypothesis about residence and the severity of cognitive deficiency.

The retrospective analysis of 152 patients hospitalized between 2021 and 2023 confirms the very high prevalence of alcohol-linked cognitive deficiency at the moment of admission (94.1% of patients had MMSE scores below 24). The magnitude of the initial deficit differs significantly depending on residence: patients from rural areas had lower average MMSE scores than those from urban areas (22.6 ± 4.3 vs. 24.8 ± 3.6; *p* = 0.02), validating our hypothesis regarding increased vulnerability among rural populations. This discrepancy suggests late consultations, reduced educational levels, and limited access to neurocognitive screening services in rural areas, a phenomenon previously reported also in Asian and European multicenter studies [46,47].

Cognitive improvement during hospitalization was modest but statistically significant across the entire lot: (+1.6 MMSE points; *p* < 0.001), reflecting the benefit of integrated interventions (sobering up, metabolic optimization, and psychotherapy). Although both rural and urban groups gained approximately 3 points at discharge, rural patients remained more frequently under the normality threshold, indicating a “partial recovery”, not a complete one. This finding corroborates with data from recent literature, which shows that alcohol-induced cognitive deficits remain only partially after short hospital interventions, and rehabilitation ambulatory programs are needed [22,36].

The initial severity of the cognitive deficit correlated in reverse with hepatic cytolysis markers, especially GGT (r = −0.41; *p* < 0.001), suggesting that chronic hepatic illness amplifies the alcohol’s neurotoxicity. This connection supports the dual pathophysiology model—hepatogenic and neurodegenerative—postulated in the last meta-analysis [23,28]. Although the correlations were comparable in both residence areas, higher GGT pathologic values in rural areas can be a supplemental factor, explaining the lower MMSE scores at admission.

As a whole, our results confirm the hypothesis of a more severe initial cognitive deficit in rural areas, and it crowns the hypothesis that residence remains an independent determinant of the MMSE score, even after adjusting for hepatic and age parameters. The persistence of this gap at discharge underlines the need for public health policies oriented towards equal access to screening services, precocious interventions, and cognitive rehabilitation programs in rural communities.

- Comparison with Literature

The findings related to patients hospitalized for chronic alcohol consumption—94% of whom presented with MMSE scores below 24 at admission—are consistent with international literature linking prolonged alcohol misuse to elevated risk of cognitive impairment and alcohol-related dementia. A systematic review obtained on European populations shows that high-risk drinking (≥24 g alcohol/day) increases the risk of dementia by 3.2–7.8% among 45–64-year-old persons, compared to those abstaining/moderately consuming—an increase order comparable to the severe prevalence found in our study, which can be explained by the hospital selection of severely chronic consumers [50].

### 4.1. Rural–Urban Disparities

We identified initial MMSE scores significantly lower in patients from rural residences. This direction is confirmed via recent trans-cultural studies: in a Chinese community sample, rural residence increased the probability of subjective cognitive decline by 48% after adjusting for sociodemographic co-variables [51], and a global synthesis concluded that the AD/ADRD incidence is systematically higher in rural areas, attributing this phenomenon to a reduced access to care, education and prevention programs. The magnitude of the MMSE gap we noticed (=2.2 points) is more modest than the 3–5 point differences reported in community cohorts, probably because our evaluation was carried out in a hospital where all patients receive sobering up and similar treatments. These findings, while statistically significant, should be interpreted within the limitations of a single-center design and in the absence of deeper contextual socioeconomic variables.

### 4.2. Hepatic Biomarkers and Cognition

The strong reversed correlation between GGT and the MMSE score (r = −0.41) replicated in our study resonates with the NHANES 2011–2014 data, where each GGT increase was associated with a deterioration of performance on the Animal Fluency test (OR = 1.16) and a non-linear relation between hepatic enzymes and a global risk of cognitive deficit [52]. This convergence strengthens the hypothesis of the “liver–brain axis”, according to which hepatic oxidative stress and hyperammonia can amplify ethanol’s neurotoxicity.

### 4.3. Consumption Intensity and Non-Linear Model

As far as consumption level is concerned, our study included exclusively patients with severe abuse, which explains the lack of any “protective factor” of small alcohol doses, suggested by some meta-analyses. The NHANES analysis, reevaluated in 2024, demonstrated a “J”-sort of relation: under the threshold of ≈10 g/day, we did not notice a decrease in CERAD scores, but over this level, each supplemental alcohol unit diminished cognitive performance [37]. The difference from our cohort, dominated by hospitalized heavy consumers, supports the idea of an existing toxic threshold, above.

Which the potential vascular benefits of alcohol are surpassed by neurotoxicity.

### 4.4. Cognitive Recovery After Abstinence

The average gain of +1.6 MMSE points during hospitalization is comparable to the 1–3 point improvements reported in intensive rehab European trials, but remains under the normalization threshold, conforming observations that short-term abstinence produces only partial recovery and that cognitive rehabilitation ambulatory programs are necessary for a complete delivery.

To synthesize our data,

○ Support the literature linking chronic alcohol abuse with severe cognitive deterioration;
○ Confirm the supplemental vulnerability of the rural residents;
○ Reproduce the negative association between GGT and cognitive function;
○ Break away from the studies suggesting benefits of moderate consumption, a discrepancy that can be explained by the severe consumption in our sample.

These convergences and differences underline the importance of layering on residence and on the degree of exposure to alcohol in future research, as well as the need to integrate hepatic biomarkers as predictors of cognitive evolution in patients with alcohol related disorders.

### 4.5. Study Limitations

While this study provides important insights into the relationship between chronic alcohol consumption and cognitive decline in older individuals, several limitations must be considered before extrapolating its findings.

First, the retrospective and single-center design, based exclusively on admission records from the “Elisabeta Doamna” Clinical Psychiatric Hospital in Galați (2021–2023), limits the ability to establish a definitive causal relationship between alcohol exposure and cognitive deterioration. The study population—hospitalized patients with severe, chronic alcohol use and delayed clinical presentation—differs substantially from the general older adults’ population, thereby restricting the external validity of the findings.

Moreover, the quality and completeness of the data varied. Although MMSE scores and basic hepatic markers were consistently recorded, several critical variables were frequently missing, including the exact duration of alcohol use, type of alcoholic beverage consumed, educational level, socioeconomic status, comorbid psychiatric or neurological conditions, and the severity of thiamine deficiency. This documentation heterogeneity may introduce classification bias and reduce the precision of the statistical estimates.

The rural–urban differences observed in MMSE scores at admission may reflect deeper disparities in healthcare access, health literacy, and socioeconomic factors that were not fully captured in this study. The analysis relied solely on MMSE and basic demographic data and did not include contextual variables such as education, income, or health-seeking behaviors. As a single-center study, the results should be interpreted as exploratory and not generalizable to all rural and urban older populations.

Confounding factors such as malnutrition, viral hepatitis, use of psychoactive substances, and polypharmacy with psychotropic medications were not controlled for in the statistical models, which included adjustments only for age, residence, and selected hepatic biomarkers.

The absence of a comparison group of moderate or abstinent drinkers may have inflated the observed prevalence of cognitive impairment and precluded the detection of any potential protective effects of low-to-moderate alcohol intake reported in other studies.

Furthermore, the MMSE was administered within the first 24 h of hospitalization, a period during which patients may have experienced acute withdrawal, delirium tremens, or sedation. This timing may have underestimated actual cognitive performance. Additionally, the MMSE is sensitive to educational background, and the lack of inter-rater calibration protocol between assessors introduces potential scoring variability. This methodological limitation was also acknowledged in the Methods section.

The short observation window (typically 10–14 days of hospitalization) prevented any assessment of medium- or long-term cognitive recovery or relapse risk. Subgroup analyses by sex, age ≥ 75, or biomarker combinations yielded limited power and wide confidence intervals.

Alcohol intake during hospitalization could not be objectively monitored; however, due to the inpatient detoxification protocol and strict supervision within the psychiatric ward, continued consumption during admission was considered unlikely.

Finally, the lack of neuroinflammatory serum markers or imaging data (e.g., brain MRI) restricted the exploration of underlying pathogenic mechanisms, which remain largely speculative.

Since this study was conducted in a psychiatric hospital, cognitive outcomes may have been influenced by psychiatric comorbidities or pharmacological interventions. Furthermore, the absence of a control group of non-drinking older adults limits the generalizability of the findings beyond this specific clinical population.

Data processing was performed using Excel, Python, and R; however, no multiple imputation was applied for missing values, and no advanced statistical modeling was used, which may predispose to overfitting or biased effect estimation.

Overall, these limitations indicate that the results should be interpreted with caution, as exploratory findings that generate hypotheses. Future research should adopt a prospective, multicenter design with broader community-based sampling, standardized data collection, and longer-term follow-up to clarify causal relationships and identify modifiable risk factors for cognitive prevention and rehabilitation.

### 4.6. Clinical Implications and Recommendations

The data obtained confirm that the majority of older chronic alcohol consumers already present a moderate–severe cognitive deficiency, and the recovery during hospitalization remains partial. In consequence, the clinical interventions must shift from the late treatment zone towards the early detection and secondary prevention zone, insisting on rural areas where we identified the most pronounced deficit.

From a practical point of view, we suggest a screening integrated pack that combines rapid cognitive evaluation through the MMSE with a routine hepatic panel (AST, ALT, bilirubin, and especially GGT). The MMSE remains the most studied instrument and the easiest to apply at the patient’s bed [53,54], and hepatology and addiction organizations already recommend the dosage of GGT to identify excessive consumption and withdrawal monitoring. The simultaneous use of the two sets of tests allows clinicians to rapidly correlate cognitive deficit severity with the potential hepatic injury and to customize the rehab intensity or the need for inter-disciplinary consultations.

In psychiatric and geriatric hospitals, the MMSE and the hepatic panel should be standard at admission, at discharge, and at the 3–6-month follow-up, in order to dynamically observe cognitive remission and hepatic function evolution. In ambulatory settings, we recommend for all patients aged ≥55-years old with alcohol consumption over the AUDIT-C threshold to receive annual MMSE and hepatic tests, according to screening principles in family doctors’ attention. The implementation is sustainable: MMSE only takes under 10 min, and hepatic panel’s cost is low, but it gives us an objective marker (GGT) both on consumption and on prognosis [55,56,57].

For rural areas, where access to a specialist is limited and where our cohort registered lower MMSE scores, telemedicine and mobile units become essential. Video platforms validated for long-distance cognitive testing proved feasibility and reliability in so-called “medical deserts” zones, reducing the diagnosis gap [58]. Integrating these solutions with teams of community assistants and with family medicine cabinets would allow repeating MMSE at home, automatically sending scores online, and alerting specialists when a decrease exceeds the established thresholds.

In addition, hepatic and cognitive screening must be accompanied by consumption reduction short interventions (brief intervention) and, where needed, by recommendation to an addiction service, in line with NIAAA recommendations and the EASL policy declaration underlining the role of early spotting of consumers at risk and asymptomatic liver [53,54,59,60]. For rural communities, the prevention strategy should be completed with local educational campaigns, stimulating the formation of non-medical sanitary staff (nurses and sanitary mediators) to administer the AUDIT-C and MMSE, as well as through thiamine supply programs and nutrition counselling to address the associated malnutrition.

Finally, public health policies can amplify clinical impact by adopting macro measures recommended by the EASL (for example, minimum price for a unit of alcohol) and by financing integrated clinical directions that include psychiatry, hepatology, neuro-psychology, and social assistance. Thus, MMSE evaluation at each medical contact and periodic dosing of GGT would not be just simple, isolated procedures, but they would be part of a continuous algorithm of screening–diagnosis–intervention–follow-up, adapted to rural and urban realities, capable of reducing the accumulated burden of alcohol-related cognitive deficiency in Romania.

## 5. Conclusions

This retrospective study that included 152 patients, ≥55 years old, hospitalized at the “Elisabeta Doamna” Clinical Psychiatric Hospital between 2021 and 2023, confirms the substantial burden of cognitive deficiency associated with chronic alcohol consumption: at admission, 94% of participants had MMSE scores below the normality threshold (<24). During hospitalization, the MMSE score went up by a median of 1.6 points (*p* < 0.001), indicating the fact that a component of cognitive deterioration is potentially reversible by managing withdrawal, correcting nutritional deficit, and improving hepatic function.

Rural–urban disparities remain evident: patients from rural residence, which represented 63.8% of the cohort, had significantly lower MMSE scores at admission than those in urban residence (22.6 vs. 24.8; *p* = 0.02), suggesting limited access to prevention services, late diagnosis, and unfavorable socioeconomic influences. At the same time, the moderate reversed correlation we observed between GGT levels and cognitive performance (r = −0.41) supports the existence of a hepatic–cerebral axis, where metabolic dysfunction and hepatic oxidative stress emphasize alcohol-induced cognitive deficit.

From a clinical point of view, the results plead for the systematic introduction, at the initial evaluation of older adults alcohol consumers, of a double screening—MMSE associated with a basic hepatic panel (AST, ALT, GGT, bilirubin, and ammonia)—for a precocious differentiation between irreversible dementia and potentially reversible hepatic encephalopathy. For the rural residents, implementing cognitive screening mobile programs, hepatic-nutritional counselling, and psycho-social support could soften the identified disparities. Also, reevaluating MMSE scores 3–6 months after discharge, integrated in a multi-disciplinary care program, would strengthen the improvements gained in the hospital and would help maintain alcohol abstinence.

The inherent limitations of the single-center, retrospective design and the absence of a control group limit generalizing our conclusions. Multi-center, prospective studies that include research on inflammatory biomarkers and neuro-structural imagery are necessary to find out the pathogenetic mechanisms of alcohol-related cognitive deficiency and to validate clinical prediction algorithms. On the whole, the data underlines the need for an integrated approach—psychiatry, gastro-hepatology, and community medicine—oriented towards a precocious screening and a comprehensive management of cognitive deficiency in old alcohol consumers, with priority for vulnerable rural populations.

## Figures and Tables

**Table 1:** MMSE scores by residence.

Variable	Rural (N = 97) M ± SD	Urban (N = 55) M ± SD	T(DF)	*p*-Value
**MMSE at admission**	22.6 ± 3.9	24.8 ± 4.2	238	2
**MMSE at discharge**	25.0 ± 3.7	25.2 ± 3.5	15	88
**MMSE improvement (δ)**	2.4 ± 1.9	2.3 ± 2.1	18	86

**Table 2:** Correlation between MMSE and hepatic markers.

Hepatic Parameter	Pearson R	*p*-Value
**TGP**	−0.26	0.0013
**TGO**	−0.29	0.0003
**GGT**	−0.41	<0.001
**Total bilirubin**	−0.06	0.47
**Ammonia**	−0.06	0.50

## Data Availability

This paper is part of the doctoral study of Psychologist Simona-Dana Mitincu-Caramfil: “The role of ethanolic consumption in depressive syndrome development”.

## Abbreviations

The following abbreviations are used in this manuscript:

MMSE	Mini-Mental State Examination
TGO	Aspartate transaminase (AST)
TGP	Alanine transaminase (ALT)
GGT	Gamma-glutamyl transferase (GGT)
NIAAA	National Institute on Alcohol Abuse and Alcoholism
EASL	The European Association for the Study of the Liver
